# Remote exercise snacking and fall-related functional outcomes in older adults: a systematic review including a meta-analysis

**DOI:** 10.3389/fphys.2026.1709619

**Published:** 2026-02-11

**Authors:** Simin Zhang, Mingkuai Wang, Ruting Lin, Zhenyu Shuai, Zeping Lv, Chu Wang, Ran Zhang, Tian Yang, Yubo Wang, Xuemin Zhang

**Affiliations:** 1 National Research Center for Rehabilitation Technical Aids, Beijing, China; 2 College of Education, Beijing Sport University, Beijing, China; 3 Physical Education Department, College of Basic Education, Beijing College of Finance and Commerce, Beijing, China

**Keywords:** exercise snack, fall-related, meta-analysis, older adults, remote

## Abstract

**Background:**

Falls are a leading cause of injury and death among older adults, yet many encounter barriers to engaging in conventional exercise programs. Remote exercise snacking (ES) refers to performing multiple (≥2 times) short bursts (≤10 min) of exercise of any type or intensity daily in a non-laboratory setting (including multiple sets of interval training), with complete rest or at least a 30-min recovery period between each exercise session, this represents a flexible alternative; however, its effectiveness remains inconclusive. This study addresses an important evidence gap by systematically evaluates the impact of remote exercise snacking on lower-limb muscle performance, balance ability, as well as its acceptability and feasibility in older adults.

**Methods:**

A systematic search was conducted in six databases (CINAHL, PubMed, Scopus, Cochrane Library, Web of Science and FMRS) from inception to May, 2025. Two reviewers independently performed study selection, data extraction, and risk of bias assessment following PRISMA guidelines. Studies meet the following eligibility criteria in accordance with PICOS, participants were insufficiently active older adults; intervention involved short bouts of exercise; comparator/control were no specific intervention; the primary outcomes was lower-limb muscle function, with secondary outcomes included balance and/or participant adherence or acceptbility; and study design were randomized crossover or randomized control only. Muscle performance and balance outcomes were synthesized through meta-analysis using Stata v15.1 with standardized mean difference (SMD), while adherence and acceptability were evaluated narratively.

**Results:**

Four publications comprising ten studies (n = 313, M/F: 170/143) were included. Remote exercise snacking significantly improved lower-limb muscle strength (SMDpooled = 0.29, 95% CI: 0.06–0.52, p = 0.01) and endurance (SMDpooled = 0.24, 95% CI: 0.01–0.46, p = 0.04), but showed no significant effect on balance (SMDpooled = 0.04, 95% CI: −0.14–0.23, p = 0.65). Subgroup analyses showed that greater improvements in strength were observed in interventions lasting 6 weeks or longer and in those that incorporated progression strategies. The overall mean adherence across the included studies was 85%, with adherence generally higher in interventions that provided video-based guidance.

**Conclusion:**

Remote exercise snacking appears effective in improving lower-limb muscle function but shows limited impact on balance among healthy older adults. Intervention duration and the inclusion of progression are key determinants of efficacy. The delivery mode (e.g., written materials, video, or app-based platforms) and exercise type (e.g., bodyweight, Tai Chi, or combined formats) may influence the acceptability and feasibility of implementation. The main findings are summarized in a graphical abstract.

**Systematic Review Registration:**

Identifier CRD42024627584.

## Graphical Abstract

**Figure:**
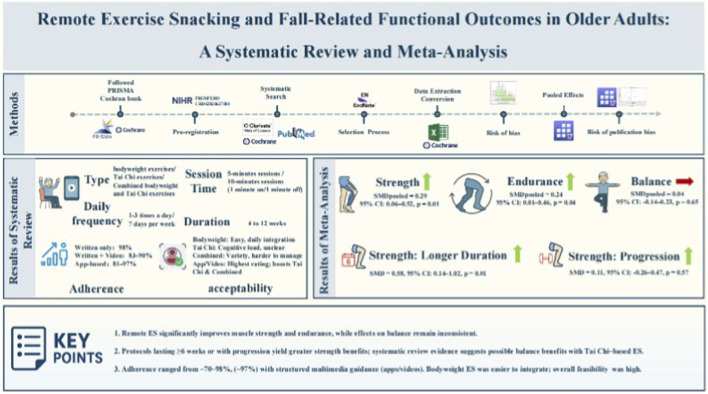

## 1 Introduction

According to the World Health Organization (WHO), 28%–35% of individuals aged 65 and older experience falls annually, with the risk rising to 32%–42% in those over 70. Falls are one of the leading causes of injury among the elderly, with 70% of them dying from falls (Mekkodathil et al., 2020; Zhu et al., 2025; W.H. Organization, 2008; James et al., 2020). Exercise interventions are recognized as one of the most effective strategies to reduce fall risk (Rikkonen et al., 2023; Zhao et al., 2017; Dautzenberg et al., 2021). A Cochrane review of 81 trials involving 19,684 participants demonstrated that exercise significantly reduces both the incidence and frequency of falls in community-dwelling older adults (Sherrington et al., 2019). However, many older individuals fail to engage in sufficient physical activity (i.e., do not meet the WHO recommendation of at least 150 min/week of moderate-intensity physical activity) due to barriers such as lack of time, low self-efficacy, and limited access to recreational facilities (Mal et al., 2025; Zhou et al., 2024; Nuzzo et al., 2024). These barriers may vary across contexts, including supervised versus unsupervised interventions, group-based versus individual exercise formats, and across countries/regions. Addressing these barriers and developing engaging, accessible exercise formats is now a critical public health priority (Bantham et al., 2021).

Remote home-based ES offers a compelling alternative to conventional structured training programs, which often require fixed schedules and physical access to fitness facilities or group classes (Nuzzo et al., 2024; Wang et al., 2025; Liang et al., 2023; Liang et al., 2022; Liang et al., 2024; Lee et al., 2024). This approach involves short, intermittent bouts of physical activity—typically around 10 min per session—performed several times throughout the day, without the need for specialized equipment (Jones et al., 2024). The integration of exercise snacking into existing remote training paradigms represents a novel approach that leverages the strengths of both models—combining the flexibility and low barrier of entry of ES with the scalability and structure of remote digital interventions (Liang et al., 2022; Fyfe et al., 2022). Compared with conventional structured training programs, remote ES may place fewer demands on time and facility access and can be more readily embedded into daily routines; accordingly, many remote ES protocols report relatively high adherence and acceptability among older adults, although adherence remains highly dependent on programme design and implementation. In addition, remote ES provides a feasible and scalable approach to support regular physical activity and potentially mitigate fall risk in ageing populations with declining mobility and functional capacity (Liang et al., 2023; Liang et al., 2024; Fyfe et al., 2022).

Despite the growing interest in remote exercise snacking, current evidence regarding its effectiveness remains inconclusive. For example, one study employing a 4-week, twice-daily remote exercise snacking protocol reported no significant improvements in muscle strength, while another with a similar design produced mixed results (Liang et al., 2024; Fyfe et al., 2022). One major limitation lies in the lack of consensus on key intervention parameters—such as frequency, intensity, and duration—which vary widely across studies and have yet to be systematically defined (Radaelli et al., 2025; D'Aurea et al., 2019; Dankel et al., 2017). In addition, existing interventions utilize diverse modes of remote guidance and supervision, including static materials (e.g., posters and activity logs), video demonstrations, and app-integrated feedback systems. Each of these delivery methods may influence adherence and intervention outcomes differently. In addition, the specific impact of remote supervision on user adherence and acceptability remains poorly understood (Preitschopf et al., 2025). Given these uncertainties, there is a clear need for a structured synthesis of existing evidence and a focused evaluation of intervention outcomes.

This study addresses an important evidence gap by systematically evaluating the effects of remote exercise snacking on lower-limb muscle performance in community-dwelling, healthy/functional older adults. In addition, we will synthesise evidence on intervention feasibility, acceptability, and adherence to better contextualise the effectiveness of remotely delivered exercise snacking in real-world settings. We hypothesise that remote exercise snacking improves lower-limb muscle performance but confers limited benefits for balance in healthy older adults. Subgroup analyses will explore how variations in exercise protocols—such as duration and progression—modulate these outcomes. By identifying key parameters that enhance the efficacy of remote exercise snacking, this study seeks to provide critical evidence to inform the development of future interventions aimed at improving musculoskeletal health and reducing fall risk in aging populations.

## 2 Methods

This systematic review and meta-analysis were performed according to the Preferred Reporting Items for Systematic Reviews and Meta-Analysis guideline (Supplementary Table S2) (Page et al., 2021) and registered with PROSPERO (ID CRD42024627584).

### 2.1 Data sources and search strategies

This study search strategy aimed to identify both published and unpublished or ongoing studies, without language restrictions. Two reviewers (S.Z. and M.W.) independently conducted a comprehensive literature search across six electronic databases: CINAHL, PubMed, Scopus, Cochrane Library, Web of Science and Foreign Medical Literature Retrieval Service (FMRS) covering all records from database inception to May 2025. Inter-rater agreement between the two reviewers was quantified using Cohen’s kappa (k). The search strategy was informed by prior literature and further refined to include a wide range of relevant terms and their synonyms (Wang et al., 2025; Jones et al., 2024). The final search terms included: “exercise snack”, “movement snack*”, “snacktivity”, “movement break”, “physical activity break”, “active break”, “vigorous intermittent lifestyle physical activity”, and “VILPA” (Jones et al., 2024) (The detailed search strategy is shown in Supplementary Table S1). To enhance the comprehensiveness of the review, backward and forward citation tracking of all included articles was conducted on 25 April and 10 May 2025, yielding 621 additional records (385 backward, 236 forward), which were included in the screening process.

### 2.2 Selection criteria

To be included in this systematic review, previous studies must meet the following eligibility criteria in accordance with PICOS.

#### 2.2.1 Participants

The participants were insufficiently active older adults (mean age ≥65 years) without severe health conditions that could impair their ability to participate in exercise interventions (e.g., unstable cardiovascular disease, uncontrolled arrhythmias or hypertension, or sarcopenia/frailty syndromes). Insufficiently active older adults were defined as those not meeting the current physical activity guidelines (i.e., <150 min/week of moderate-intensity aerobic activity, <75 min/week of vigorous-intensity aerobic activity, or an equivalent combination).

#### 2.2.2 Intervention

Remotely ES interventions consisting of short bouts of exercise (15 s–10 min each) using bodyweight or resistance exercises, performed under supervised or unsupervised conditions (e.g., via video, mobile app, booklet, or exergame). These bouts could be repeated multiple times per day (totaling about 60 min or more).

#### 2.2.3 Comparator/control

No specific intervention, maintaining usual daily habits, or receiving physical activity education (e.g., habitual behavior, usual physical activity, or no structured exercise).

#### 2.2.4 Outcomes

The primary outcome was defined as a measure of lower-limb muscle function (e.g., muscle strength or power). Secondary outcomes included measures of balance/mobility (e.g., timed up-and-go) and participant adherence to, and acceptability of, the ES intervention.

#### 2.2.5 Study design

The design of the study was a randomized crossover or randomized control only.

Articles were excluded if they fulfilled the following criteria: 1) animal trials; 2) unable to obtain outcome data; 3) review papers and conference articles; and 4) repeated publications.

### 2.3 Data extraction and outcomes

The process of data extraction was conducted according to the Cochrane Collaboration Handbook. Two authors (M.W and S. Z) independently performed data extraction, and when a decision disagreement happened, it was discussed with the third author (X.Z) until a consensus was achieved. The extracted information from the publications included: the study (authors, year), participants (age, height, weight, sex, and eligibility criteria), grouping and sample size, ES interventions (frequency, intensity, time, type, duration), and outcome measures (Li et al., 2019).

Most studies only report data for pre- and post-intervention. Thus, average change was calculated as the difference between the mean of data pre- and post-intervention. The specific formula can be found in the Supplementary Material.

### 2.4 Quality assessment

Two reviewers independently assessed the risk of bias, resolving disagreements through discussion when possible or, if necessary, by arbitration from a third researcher, using the Cochrane Collaboration’s Risk of Bias Tool 2 (ROB 2) (Sterne et al., 2019). This tool evaluates five domains: random sequence generation, random allocation concealment, blinding of outcome assessment, incomplete outcome data, and selective outcome reporting.

### 2.5 Statistical analysis

Standardized mean difference (SMD, Hedge’s g) with 95% confidence interval (CI) was used to assess the effect size. Effect size was classified as trivial (<0.2), small (0.2–0.49), moderate (0.5–0.79), or large (>0.8) (Cohen, 2013). Meta-analysis was performed in Stata v15.1 (STATA Corp., College Station, TX) using the inverse-variance method. The I^2^ statistic was used to evaluate heterogeneity among the trials with the following criteria: trivial (<25%), low (25–50%), moderate (50–75%), and high (>75%) (Hi et al., 2003). A random-effects model was used to estimate pooled effects, as heterogeneity was anticipated across studies due to differences in participants and interventions. Subgroup analysis was used to explore potential sources of heterogeneity (Bandeira-Guimarães et al., 2023). The Funnel plots and Egger tests were used to evaluate publication bias. If potential publication bias was detected, we used the trim and fill method for the sensitivity analysis of the results (Duval and Tweedie, 2000). All the statistical significance was set at a p-value of <0.05.

## 3 Results

### 3.1 Study selection

**FIGURE 1:**
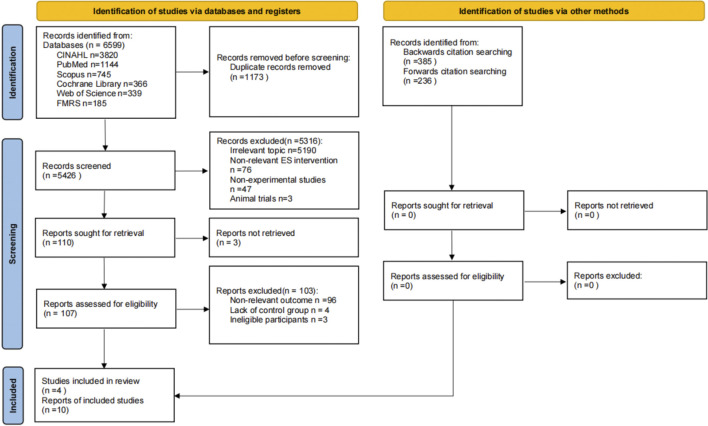
Study flowchart.

The study selection process is summarized in Figure 1. A total of 6599 records were identified through six electronic databases: CINAHL (n = 3820), PubMed (n = 1144), Scopus (n = 745), Cochrane Library (n = 366), Web of Science (n = 339), and FMRS (n = 185). After removing 1173 duplicate entries, 5426 records remained for title and abstract screening. Following the screening phase, 5316 records were excluded due to irrelevance. A total of 110 full-text reports were assessed for eligibility, of which 3 were unavailable because the authors have not been reached, leaving 103 that were excluded based on the following criteria: irrelevant results (n = 96), lack of control group (n = 4), and ineligible participants (n = 3).

Ultimately, 4 publications comprising 10 individual studies met the inclusion criteria and were included in the systematic review. Inter-rater agreement was very high (Cohen’s κ = 0.85). All 10 studies were also eligible for quantitative synthesis (see Table 1 for study characteristics). Notably, three of the included publications (Liang et al., 2022; Liang et al., 2024; Fyfe et al., 2022; Perkin et al., 2019) each reported data from multiple randomized controlled trials (RCTs), contributing to the total of 10 included studies.

**TABLE 1:** Characteristics of the included studies (n = 10).

N	Study	Study type setting	Participants N, M/F	Age; BMI	Eligibility criteria	Exercise snack group (frequency, intensity, time, type)	Adherence	Acceptability	Control group	Outcome
1	Perkin et al. (2019)	RCT	N = 20 6/14	72 ± 5 years 26 ± 3 kg/m^2^	Community-dwelling older adults who were physically inactive but otherwise healthy	F: 2/day	Adherence to the ES intervention was 98%, with participants completing an average of 54 out of 56 sessions	—	Usual activity	Peak force → 60-s STS (N) ↑
						I: As many as possible				
						T: 10 min (1 min on/1 min off)				
						T: 5 bodyweight exercises				
						Duration: 4 weeks				
2	Liang et al. (2022) (Exp.1)	RCT	N = 30 20/10	72 ± 5 years NR	Older adults who were physically inactive but otherwise healthy (no chronic disease)	F: 2/day, 7 days/week	1. Mean days attempted (out of 28): 26 ± 3 2. Percentage adherence: 90%	Rated as most acceptable, with clear instructions and easy tracking. Some participants found it “boring” due to simplicity	Physical activity Advice	5-Repetition STS → 60-s STS (N) → R leg standing balance → L leg standing balance →
						I: As many as possible				
						T: 5 min (1 min on/1 min off)				
						T: 5 bodyweight exercises				
						Duration: 4 weeks				
3	Liang et al. (2022) (Exp.2)	RCT	N = 29 22/7	72 ± 5 years NR	Older adults who were physically inactive but otherwise healthy (no chronic disease)	F: 2/day, 7 days/week	1. Mean days attempted (out of 28): 26 ± 6 2. Percentage adherence: 84%	Acceptability lower than ES. Feedback highlighted unclear instructions and frustration with difficulty performing exercises	Physical activity Advice	5-Repetition STS → 60-s STS (N) → R leg standing balance → L leg standing balance →
						I: As many as possible				
						T: 5 min (1 min on/1 min off)				
						T: 5 Tai Chi exercises				
						Duration: 4 weeks				
4	Liang et al. (2022) (Exp.3)	RCT	N = 29 19/10	72 ± 5 years NR	Older adults who were physically inactive but otherwise healthy (no chronic disease)	F: 2/day, 7 days/week	1. Mean days attempted (out of 28): 26 ± 4 2. Percentage adherence: 83%	Acceptability moderate. Participants appreciated variety but found managing both components challenging	Physical activity Advice	5-Repetition STS → 60-s STS (N) → R leg standing balance → L leg standing balance →
						I: As many as possible				
						T: 10 min (1 min on/1 min off)				
						T: 5 bodyweight exercises combination 5 Tai Chi exercises				
						Duration: 4 weeks				
5	Fyfe et al. (2022) (Exp.1)	RCT	N = 19 7/12	70 ± 4 years 27 ± 5 kg/m^2^	Community-dwelling older adults who were physically and cognitively able and not engaged in regular structured exercise	F: 1/day, 7 days/week	1. Mean adherence: 97% 2. High adherence level with minimal adverse incidents reported	1. Rated highly for ease and integration into routines 2. Requests for more variety and upper-body exercises	Usual activity	5-Repetition STS → 30-s STS (N) →
						I: As many as possible				
						T: 10 min (1 min on/1 min off)				
						T: 5 multi-joint bodyweight exercises				
						Duration: 4 weeks				
						Other: Remotely delivered via an app				
6	Fyfe et al. (2022) (Exp.2)	RCT	N = 20 8/12	70 ± 4 years 27 ± 5 kg/m^2^	Community-dwelling older adults who were physically and cognitively able and not engaged in regular structured exercise	F: 2/day, 7 days/week	1. Mean adherence: 82% 2. Moderate adherence level, with minor musculoskeletal complaints reported but manageable adjustments made to continue participation	1. Appreciated balance of frequency and duration 2. Recommendations for more variety and tailored exercises	Usual activity	5-Repetition STS → 30-s STS (N) →
						I: As many as possible				
						T: 10 min (1 min on/1 min off)				
						T: 5 multi-joint bodyweight exercises				
						Duration: 4 weeks				
7	Fyfe et al. (2022) (Exp.3)	RCT	N = 19 7/12	70 ± 4 years 27 ± 5 kg/m^2^	Community-dwelling older adults who were physically and cognitively able and not engaged in regular structured exercise	F: 3/day, 7 days/week	1. Mean adherence: 81% 2. Similar adherence level to twice-daily, with participants continuing despite minor musculoskeletal complaints	1. Frequent sessions valued but harder to fit into routines 2. Suggestions for adding variety and external loads	Usual activity	5-Repetition STS → 30-s STS (N) →
						I: As many as possible				
						T: 10 min (1 min on/1 min off)				
						T: 5 multi-joint bodyweight exercises				
						Duration: 4 weeks				
8	Liang et al. (2024) (Exp.1)	RCT	N = 49 27/22	74.1 ± 5.5 years; NR	65 years of age or older and were not engaging in regular sport or exercise	F: 2 sessions/day, 7 days/week	A total of 27 participants were assigned to the intervention group. Five participants stopped exercising and withdrew from the study after a total of 4 weeks, resulting in an adherence rate of 81.5%	1. Acceptability score: 4.21 ± 0.34 2. Participants noted increased confidence and ability in daily tasks; Tai chi favored for its relaxing nature, though cognitively demanding for some; Highlighted the need for more guidance or tailored options to improve adherence and efficacy	Normal habitual Behaviour	5 repetition STS ↑ 60-s STS (N) → R Leg standing balance ↑ L Leg standing balance →
						I: As many as possible				
						T: 5 min (1 min on/1 min off)/10 min (1 min on/1 min off)				
						T: 5 bodyweight exercises, 5 Tai chi exercises, or combination of both				
						Duration: 4 weeks				
9	Liang et al. (2024) (Exp.2)	RCT	N = 49 27/22	74.1 ± 5.5 years NR	65 years of age or older and were not engaging in regular sport or exercise	F: 2 sessions/day, 7 days/week	A total of 27 participants were assigned to the intervention group. Six participants stopped exercising and withdrew from the study after a total of 8 weeks, resulting in an adherence rate of 77.8%	1. Acceptability score: 4.21 ± 0.34 2. Participants noted increased confidence and ability in daily tasks; Tai Chi favored for its relaxing nature, though cognitively demanding for some; Highlighted the need for more guidance or tailored options to improve adherence and efficacy	Normal habitual behaviour	5 repetition STS ↑ 60-s STS (N) → R leg standing balance ↑ L leg standing balance ↑
						I: As many as possible				
						T: 5 min (1 min on/1 min off)/10 min (1 min on/1 min off)				
						T: 5 bodyweight exercises, 5 Tai Chi exercises, or combination of both				
						Duration: 8 weeks				
10	Liang et al. (2024) (Exp.3)	RCT	N = 49 27/22	74.1 ± 5.5 years NR	65 years of age or older and were not engaging in regular sport or exercise	F: 7 sessions/day, 3 days/week	A total of 27 participants were assigned to the intervention group. Five participants stopped exercising and withdrew from the study after a total of 12 weeks, resulting in an adherence rate of 77.8%	1. Acceptability score: 4.08 ± 0.31 2 Most participants expressed satisfaction and motivation; Recognized physical improvements, including strength, balance, and flexibility; High willingness to continue or extend the program’s duration and intensity	Normal habitual behaviour	5 repetition STS → 60-s STS (N) → R leg standing balance → L leg standing balance →
						I: As many as possible				
						T: 5 min (1 min on/1 min off)/10 min (1 min on/1 min off)				
						T: 5 bodyweight exercises, 5 Tai Chi exercises, or combination of both				
						Duration: 12 weeks				

N	Study	Term	Intervention details
1	Perkin et al. (2019)	Exercise snacking	Sit-to-stand, seated knee extensions, standing knee bends, marching on the spot, standing calf raises
2	Liang et al. (2022) (Exp.1)	Exercise snacking	Five movements (sit-to-stand from a chair, seated knee extensions of alternating legs, standing knee bends of alternating legs, marching on the spot, and standing calf raises)
3	Liang et al. (2022) (Exp.2)	Tai chi snacking	Five chen style tai chi movements (cloud hands, going left, stand on one leg, single whip, snake creeps through the grass, and front heel kick)
4	Liang et al. (2022) (Exp.3)	Combination	Participants were instructed to do one exercise snacking bout and one tai chi snacking bout (as described above)
5	Fyfe et al. (2022) (Exp.1)	Resistance exercise snacking	Once for 4 weeks. For weeks 1–2, the prescribed exercises were: 1) chair sit-to-stand (no arms), 2) single-leg quarter squat (with chair support), 3) side lunges, 4) calf raise (with chair support), and 5) clock stepping (with chair support). To provide variety and progressive overload, the five exercises prescribed for weeks 3–4 were changed to: 1) squat into high knee march (knee to elbow), 2) single-leg quarter squat, 3) rapid step ups (involving a rapid concentric phase and performed using a staircase step, or another available step of a similar height), 4) single-leg calf raise, and 5) rapid clock stepping
6	Fyfe et al. (2022) (Exp.2)	Resistance exercise snacking	Twice for 4 weeks. For weeks 1–2, the prescribed exercises were: 1) chair sit-to-stand (no arms), 2) single-leg quarter squat (with chair support), 3) side lunges, 4) calf raise (with chair support), and 5) clock stepping (with chair support). To provide variety and progressive overload, the five exercises prescribed for weeks 3–4 were changed to: 1) squat into high knee march (knee to elbow), 2) single-leg quarter squat, 3) rapid step ups (involving a rapid concentric phase and performed using a staircase step, or another available step of a similar height), 4) single-leg calf raise, and 5) rapid clock stepping
7	Fyfe et al. (2022) (Exp.3)	Resistance exercise snacking	Thrice for 4 weeks. For weeks 1–2, the prescribed exercises were: 1) chair sit-to-stand (no arms), 2) single-leg quarter squat (with chair support), 3) side lunges, 4) calf raise (with chair support), and 5) clock stepping (with chair support). To provide variety and progressive overload, the five exercises prescribed for weeks 3–4 were changed to: 1) squat into high knee march (knee to elbow), 2) single-leg quarter squat, 3) rapid step ups (involving a rapid concentric phase and performed using a staircase step, or another available step of a similar height), 4) single-leg calf raise, and 5) rapid clock stepping

### 3.2 Characteristics of included studies

#### 3.2.1 Participant characteristics

A total of 313 participants across 10 studies were included in the analysis, comprising 170 males (54.3%) and 143 females (45.7%). Participants were older adults aged between 65 and 75 years. BMI data were reported in four studies, ranging from 26 ± 3 kg/m^2^ to 27 ± 5 kg/m^2^, while six studies did not provide BMI information.

#### 3.2.2 ES protocol

The four publications and ten included studies shared a consensus on the basic principles of ES intervention but showed variations in intervention type, duration, and frequency. Five studies (Perkin et al., 2019; Liang et al., 2022 Exp.1; Fyfe et al., 2022 Exp.1; Fyfe et al., 2022 Exp.2; Fyfe et al., 2022 Exp.3) employed multi-joint bodyweight exercises (Liang et al., 2022; Fyfe et al., 2022; Perkin et al., 2019), one (Liang et al., 2022 Exp.2) study used simplified Tai Chi exercises (Liang et al., 2022), and four studies (Liang et al., 2022 Exp.3; Liang et al., 2024 Exp.1; Liang et al., 2024 Exp.2; Liang et al., 2024 Exp.3) used a combination of bodyweight and Tai Chi exercises (Liang et al., 2022; Liang et al., 2024).

In terms of session time, eight studies (Perkin et al., 2019; Liang et al., 2022 Exp.3; Fyfe et al., 2022 Exp.1; Fyfe et al., 2022 Exp.2; Fyfe et al., 2022 Exp.3; Liang et al., 2024 Exp.1; Liang et al., 2024 Exp.2; Liang et al., 2024 Exp.3) implemented 10-min sessions (1 min on/1 min off) (Liang et al., 2022; Liang et al., 2024; Fyfe et al., 2022; Perkin et al., 2019), while two studies (Liang et al., 2022 Exp.1; Liang et al., 2022 Exp.2) used 5-min sessions (1 min on/1 min off) (Liang et al., 2022). The intensity followed the “as many as possible” standard.

The duration of ES interventions ranged from 4 to 12 weeks. Eight studies (Perkin et al., 2019; Liang et al., 2022 Exp.1; Liang et al., 2022 Exp.2; Liang et al., 2022 Exp.3; Fyfe et al., 2022 Exp.1; Fyfe et al., 2022 Exp.2; Fyfe et al., 2022 Exp.3; Liang et al., 2024 Exp.1) used a 4-week program (Liang et al., 2022; Liang et al., 2024; Fyfe et al., 2022; Perkin et al., 2019), one (Liang et al., 2024 Exp.2) used an 8-week program (Liang et al., 2024), and one (Liang et al., 2024 Exp.3) used a 12-week program (Liang et al., 2024). Daily intervention frequency varied: one study (Fyfe et al., 2022 Exp.1) implemented it once daily (Fyfe et al., 2022), eight studies (Perkin et al., 2019; Liang et al., 2022 Exp.1; Liang et al., 2022 Exp.2; Liang et al., 2022 Exp.3; Fyfe et al., 2022 Exp.2; Liang et al., 2024 Exp.1; Liang et al., 2024 Exp.2; Liang et al., 2024 Exp.3) twice daily (Liang et al., 2022; Liang et al., 2024; Fyfe et al., 2022; Perkin et al., 2019), and one study (Fyfe et al., 2022 Exp.3) three times daily (Fyfe et al., 2022). All ten studies delivered interventions 7 days per week (Liang et al., 2022; Liang et al., 2024; Fyfe et al., 2022; Perkin et al., 2019).

Progression was defined as the planned, systematic adjustment of training variables over time to maintain progressive overload, including increases in load/intensity and/or repetitions, as well as modifications to repetition velocity, rest intervals, total training volume, training frequency, and exercise selection or complexity (Kraemer and Ratamess, 2004; Progression Models in Resistance Training, 2009). In terms of progression of ES interventions, six studies Fyfe et al., 2022 Exp.1; Fyfe et al., 2022 Exp.2; Fyfe et al., 2022 Exp.3; Liang et al., 2024 Exp.1; Liang et al., 2024 Exp.2; Liang et al., 2024 Exp.3) implemented interventions with planned progression (Liang et al., 2024; Fyfe et al., 2022), while four studies (Perkin et al., 2019; Liang et al., 2022 Exp.1; Liang et al., 2022 Exp.2; Liang et al., 2022 Exp.3) did not report any progression (Liang et al., 2022; Perkin et al., 2019).

#### 3.2.3 Outcome measurements

The studies included in this review examined the effects of ES interventions on participants' lower limb muscle strength, endurance, and balance. For muscle strength, one study (Perkin et al., 2019) assessed strength using a pneumatic leg press dynamometer (Perkin et al., 2019), while nine studies (Liang et al., 2022 Exp.1; Liang et al., 2022 Exp.2; Liang et al., 2022 Exp.3; Fyfe et al., 2022 Exp.1; Fyfe et al., 2022 Exp.2; Fyfe et al., 2022 Exp.3; Liang et al., 2024 Exp.1; Liang et al., 2024 Exp.2; Liang et al., 2024 Exp.3) evaluated strength by measuring the time taken to complete five repetitions of the STS test (Liang et al., 2022; Liang et al., 2024; Fyfe et al., 2022). For muscle endurance, all ten studies used the 60-s or 30-s STS test to assess endurance in older adults (Liang et al., 2022; Liang et al., 2024; Fyfe et al., 2022; Perkin et al., 2019). Balance ability was assessed in six studies (Liang et al., 2022 Exp.1; Liang et al., 2022 Exp.2; Liang et al., 2022 Exp.3; Liang et al., 2024 Exp.1; Liang et al., 2024 Exp.2; Liang et al., 2024 Exp.3) by measuring the duration of single leg standing for both the left and right legs (Liang et al., 2022; Liang et al., 2024).

Secondary outcomes, adherence was measured in several ways. One study (Perkin et al., 2019) tracked the number of sessions completed out of the prescribed total (Perkin et al., 2019). Three studies (Liang et al., 2022 Exp.1–3) recorded the number of days participants attempted the exercises and compared adherence between the ES and control groups (Liang et al., 2022). Three studies (Fyfe et al., 2022 Exp.1–3) assessed adherence by tracking the number of completed sessions versus prescribed sessions, as well as the frequency of “exercise snacks” per week. For acceptability, three studies used an eight-item online questionnaire based on the Theoretical Framework of Acceptability (TFA) (Fyfe et al., 2022). Three studies (Liang et al., 2024 Exp.1–3) gathered participant feedback on the ease of integration into routines and the desire for more variety (Liang et al., 2024).

#### 3.2.4 Effects of ES on lower limb muscle performance and balance ability

Eight studies (Perkin et al., 2019; Liang et al., 2022 Exp.1; Liang et al., 2022 Exp.2; Liang et al., 2022 Exp.3; Fyfe et al., 2022 Exp.1; Fyfe et al., 2022 Exp.2; Fyfe et al., 2022 Exp.3; Liang et al., 2024 Exp.3) reported no significant improvement in lower limb muscle strength following ES interventions compared to control groups (Liang et al., 2022; Liang et al., 2024; Fyfe et al., 2022; Perkin et al., 2019), while two studies (Liang et al., 2024 Exp.1; Liang et al., 2024 Exp.2) showed significant improvements (Liang et al., 2024). Nine studies (Liang et al., 2022 Exp.1; Liang et al., 2022 Exp.2; Liang et al., 2022 Exp.3; Fyfe et al., 2022 Exp.1; Fyfe et al., 2022 Exp.2; Fyfe et al., 2022 Exp.3; Liang et al., 2024 Exp.1; Liang et al., 2024 Exp.2; Liang et al., 2024 Exp.3) found no significant improvement in lower limb muscle endurance following ES interventions compared to control groups (Liang et al., 2022; Liang et al., 2024; Fyfe et al., 2022), while one study (Perkin et al., 2019) showed a significant improvement (Perkin et al., 2019). Four studies (Liang et al., 2022 Exp.1; Liang et al., 2022 Exp.2; Liang et al., 2022 Exp.3; Liang et al., 2024 Exp.3) reported no significant improvement in lower limb balance ability following ES interventions compared to the control group (Liang et al., 2022; Liang et al., 2024), while two other studies (Liang et al., 2024 Exp.1; Liang et al., 2024 Exp.2) showed significant improvements (Liang et al., 2024).

#### 3.2.5 Adherence and acceptability of ES interventions

In terms of adherence, three studies (Perkin et al., 2019; Liang et al., 2022 Exp.1; Fyfe et al., 2022 Exp.1) demonstrated adherence rates of 90% or higher (Liang et al., 2022; Fyfe et al., 2022; Perkin et al., 2019). Five studies showed adherence rates between 80% and 90% (Liang et al., 2022; Exp.2; Liang et al., 2022; Exp.3; Fyfe et al., 2022 Exp.2; Fyfe et al., 2022 Exp.2; Liang et al., 2024 Exp.1) (Liang et al., 2022; Liang et al., 2024; Fyfe et al., 2022). Two studies reported adherence rates between 70% and 80% (Liang et al., 2024 Exp.1; Liang et al., 2024 Exp.2) (Liang et al., 2024).

Regarding acceptability, four studies (Perkin et al., 2019; Liang et al., 2022; Exp.1; Fyfe et al., 2022; Exp.1; Liang et al., 2024; Exp.1) found that participants rated the intervention as acceptable, with some requesting more variety or specific modifications, such as incorporating upper-body exercises or providing additional guidance (Liang et al., 2022; Liang et al., 2024; Fyfe et al., 2022; Perkin et al., 2019). Three studies (Liang et al., 2022; Exp.2; Liang et al., 2022; Exp.3; Fyfe et al., 2022; Exp.2) showed moderate acceptability, with participants appreciating variety but highlighting challenges related to the complexity of the exercises or balancing multiple components (Liang et al., 2022; Fyfe et al., 2022). Two studies (Liang et al., 2024; Exp.2; Liang et al., 2024; Exp.3) exhibited lower acceptability, with participants noting the need for more guidance or adjustments to improve adherence and efficacy, especially for Tai Chi exercises that were cognitively demanding for some (Liang et al., 2024).

### 3.3 Quality assessment

**FIGURE 2:**
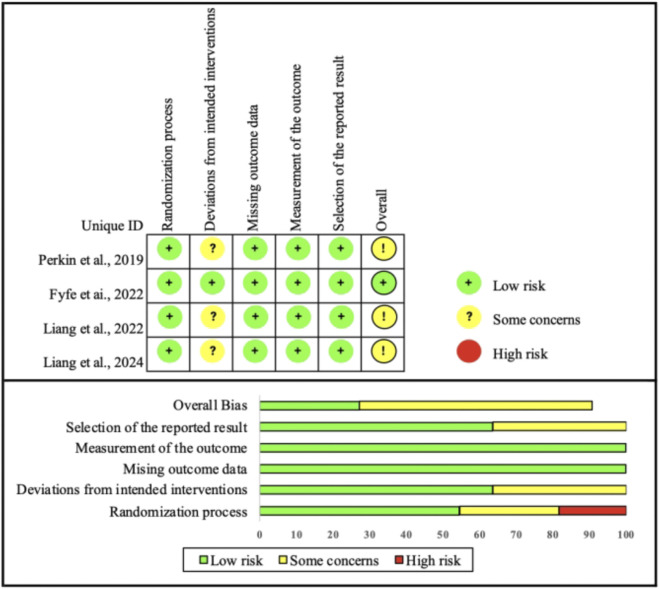
Risk of bias in the included studies.

The risk of bias assessment is shown in Figure 2. All four trials were rated as having “some concerns.” Perkin et al. (2019), Liang et al. (2022), and Liang et al. (2024) raised issues in deviations from intended interventions due to limited reporting of adherence and fidelity. Perkin et al. (2019) also showed concerns in selection of the reported result because primary outcomes were not pre-specified. All studies were judged low risk in randomization, missing data, and outcome measurement, reflecting acceptable methodological conduct. No trial was rated high risk in any domain, supporting inclusion of all studies in the meta-analysis.

### 3.4 Meta-analysis

The subgroup analysis was conducted to explore possible sources of heterogeneity, focusing on factors such as the duration of the intervention (i.e., less than 6 weeks vs. 6 weeks or longer) and the progression of the ES protocol.

#### 3.4.1 Effects of ES on lower limb muscle strength

**FIGURE 3:**
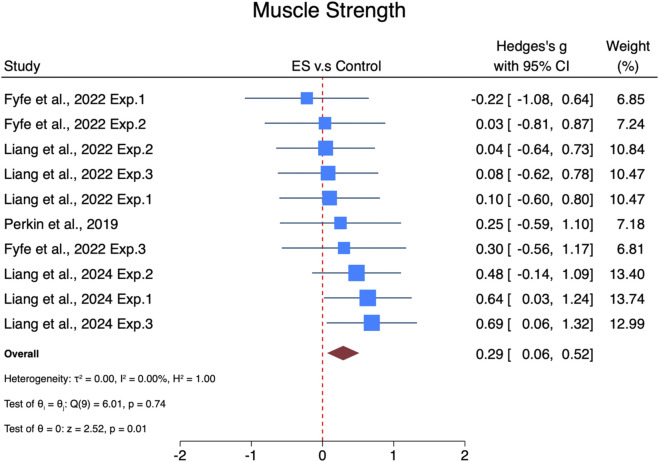
Forest plot of the effects of ES on lower limb muscle strength. Exp.1, Experiment 1; Exp.2, Experiment 2; Exp.3, Experiment 3.

The pooled effect size of ES was interventions on lower limb muscle strength small but statistically significant (SMDpooled = 0.29, 95% CI: 0.06–0.52, p = 0.01, Figure 3) and showed no heterogeneity (I^2^ = 0%, p = 0.74). The funnel plot (Supplementary Figure S1A) and Egger’s test (t = −1.65, p = 0.14) suggested no publication bias.

Subgroup analyses (Figure 6) revealed that interventions lasting less than 6 weeks had a non-significant trivial effect size (SMD = 0.19, 95% CI: −0.08–0.45, p = 0.17). In contrast, interventions lasting 6 weeks or more showed a statistically significant moderate effect size (SMD = 0.58, 95% CI: 0.14–1.02, p = 0.01). Regarding progression, interventions with progression demonstrated a significant moderate effect size (SMD = 0.41, 95% CI: 0.12–0.7, p = 0.01), while those without progression exhibited a non-significant trivial effect size (SMD = 0.11, 95% CI: −0.26–0.47, p = 0.57).

#### 3.4.2 Effects of ES on lower limb muscle endurance

**FIGURE 4:**
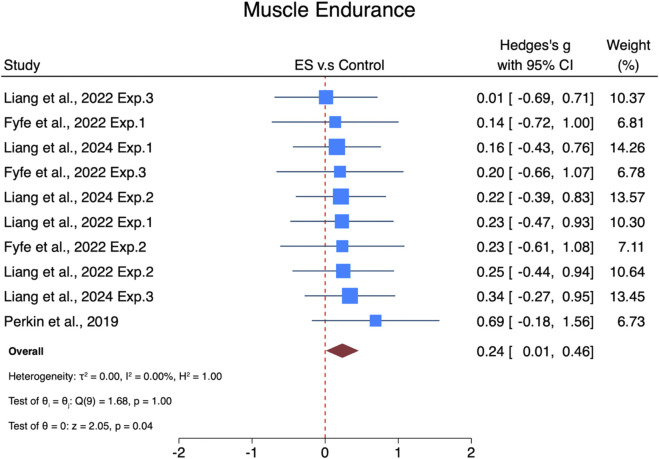
Forest plot of the effects of ES on lower limb muscle endurance. Exp.1, Experiment 1; Exp.2, Experiment 2; Exp.3, Experiment 3.

The pooled effect size of ES interventions on lower limb muscle endurance was small but statistically significant (SMDpooled = 0.24, 95% CI: 0.01–0.46, p = 0.04, Figure 4) and showed no heterogeneity (I^2^ = 0%, p = 1). The funnel plot (Supplementary Figure S1B) and Egger’s test (t = 0.33, p = 0.75) suggested no publication bias.

Subgroup analyses (Figure 6) revealed that interventions lasting less than 6 weeks had a non-significant small effect size (SMD = 0.22, 95% CI: −0.04–0.48, p = 0.1). Interventions lasting 6 weeks or more showed a non-significant small effect size (SMD = 0.28, 95% CI: −0.16–0.71, p = 0.21). Regarding progression, interventions with progression showed a non-significant small effect size (SMD = 0.22, 95% CI: −0.06–0.51, p = 0.13), while interventions without progression showed a non-significant small effect size (SMD = 0.26, 95% CI: −0.11–0.62, p = 0.17).

#### 3.4.3 Effects of ES on balance ability

**FIGURE 5:**
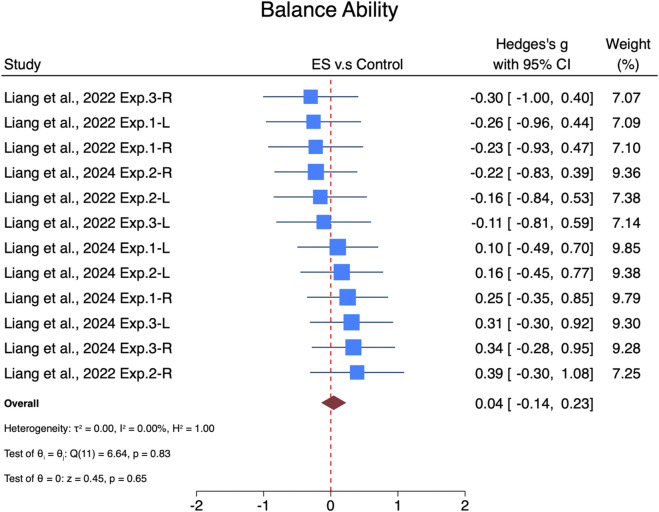
Forest plot of the effects of ES on balance ability. Exp.1, Experiment 1; Exp.2, Experiment 2; Exp.3, Experiment 3.

The pooled effect size of ES interventions on balance ability was trivial and non-significant (SMDpooled = 0.04, 95% CI: −0.14–0.23, p = 0.65, Figure 5) and showed no heterogeneity (I^2^ = 0%, p = 0.83). The funnel plot (Supplementary Figure S1C) and Egger’s test (t = −1.25, p = 0.26) suggested no publication bias.

**FIGURE 6:**
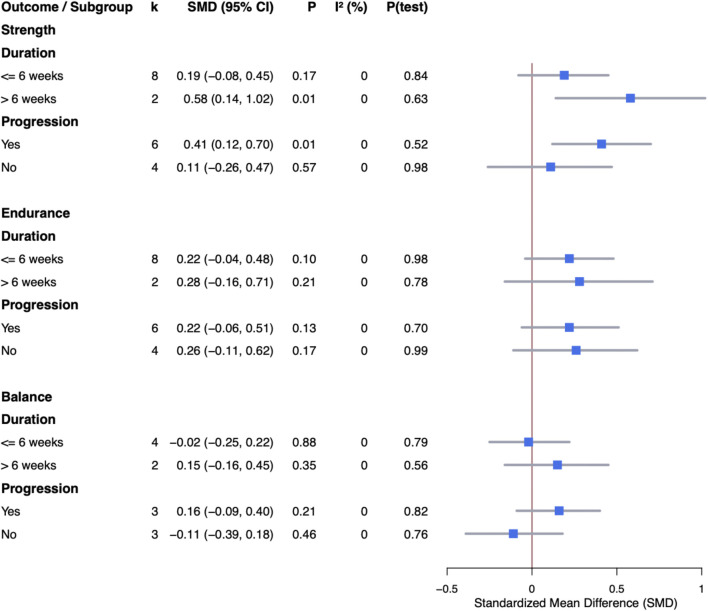
Subgroup analysis results regarding the effects of ES.

Subgroup analyses (Figure 6) revealed that interventions lasting less than 6 weeks had a non-significant trivial effect size (SMD = −0.02, 95% CI: −0.25–0.22, p = 0.88). Interventions lasting more than 6 weeks showed a non-significant trivial effect size (SMD = 0.15, 95% CI: −0.16–0.45, p = 0.35). For progression, interventions with progression showed a non-significant trivial effect size (SMD = 0.16, 95% CI: −0.09–0.40, p = 0.21), while those without progression showed a non-significant trivial effect size (SMD = −0.11, 95% CI: −0.39–0.18, p = 0.46).

## 4 Discussion

This meta-analysis is the first to systematically examine the effects of remote ES interventions on lower limb muscle performance and balance ability in healthy older adults. The findings suggest that ES protocols can enhance muscle strength and endurance of healthy older adults, but show no improvement in balance ability. Subgroup analyses revealed that key moderating factors, such as intervention duration and progression, offer valuable insights for refining future ES protocols. Based on the above, a graphical abstract summarizes the main findings of this study.

Remote ES may have potential advantages for enhancing lower limb muscle strength and endurance in healthy older adults, although the underlying mechanisms remain unclear, particularly regarding the role of resistance exercise snacking in strength development (Wang et al., 2025). Interestingly, we found that muscle strength was more significantly affected by remote ES protocols compared to muscle endurance. Specifically, protocols lasting more than 6 weeks were associated with larger improvements in muscle strength than those lasting less than 6 weeks. For example, Fyfe et al. found that a 4-week remote ES protocol with twice-daily interventions and seven sessions per week did not improve the 5-STS (Fyfe et al., 2022). In contrast, Liang et al. demonstrated that an 8-week protocol with the same frequency and duration led to significant improvements in the 5-STS (Liang et al., 2024). Progression strategies within remote ES protocols also followed a similar trend. In Liang et al.'s study, progression involved expanding the range of motion, reducing external support, transitioning to unilateral weight-bearing movements, incorporating complex arm movements, and increasing time under tension (Nuzzo et al., 2024; Liang et al., 2024; Radaelli et al., 2025; Kraemer and Ratamess, 2004). Participants selected progression levels based on their self-perceived capability. This personalized approach, implemented in a 4-week remote ES protocol with twice-daily sessions, significantly improved 5-STS performance (Liang et al., 2024). In contrast, Liang et al.'s study, using the same protocol but with a standardized, non-individualized progression approach (Liang et al., 2022), as well as Fyfe et al.'s study, which did not incorporate progression strategies, found no significant improvements in 5-STS performance (Fyfe et al., 2022).

Current remote ES protocols have yielded inconsistent results in improving balance ability. We found that short-duration ES protocols showed no improvement in balance among older adults. A potential explanation is that meaningful gains in balance may require longer-term, progressive loading and neuromuscular adaptation to enhance proprioception and postural control (Su et al., 2024; Babagoltabar-Samakoush et al., 2025; Yılmaz et al., 2024). Additionally, a study with a four-week remote ES protocol involving seven sessions per week and two sessions per day, but without Tai Chi movements, did not improve balance (Liang et al., 2022). In contrast, the same protocol with Tai Chi movements significantly enhanced balance ability (Liang et al., 2024). This effect may be attributed to Tai Chi’s controlled, slow movements and weight shifting, which promote dynamic stability and coordination (Yang et al., 2024; Wehner et al., 2021; Penn et al., 2019). However, these findings are derived from complete Tai Chi training protocols, and modified versions adapted for remote ES lack mechanistic studies. Further research is needed to explore the underlying mechanisms of remote Tai Chi-based ES interventions (Wang et al., 2025). Moreover, the observed benefits of Tai Chi ES on balance were demonstrated exclusively in healthy older adults with no balance impairments. This may obscure its potential effects in more vulnerable populations, such as frail older adults or individuals with balance deficits. Future studies should investigate the effectiveness of remote Tai Chi ES in these at-risk populations (Nuzzo et al., 2024; Wang et al., 2025; Dai et al., 2024).

This study highlights the influence of exercise modality, supervision format, and progression strategies on adherence and acceptability in remote ES protocols. Interventions incorporating structured multimedia guidance, such as video-based instructional content and mobile application supervision (e.g., PhysiTrack™ and PhysiApp), achieved high adherence rates (∼97%), suggesting that clear instructional support enhances participant engagement (Fyfe et al., 2022). Acceptability was also influenced by exercise selection and session structure. Participants generally favored bodyweight-based ES protocols, which were easier to integrate into daily routines (Perkin et al., 2019). In contrast, Tai Chi-based ES, while beneficial for some, was perceived as cognitively demanding, suggesting that cognitive load and perceived complexity may influence engagement (Liang et al., 2022; Liang et al., 2024). Future research should focus on developing remote ES protocols that enhance operability, acceptability, and adherence, while still achieving physiological benefits within established thresholds. These remote ES protocols should increasingly emphasize real-world applicability, incorporate gamification, and facilitate social interaction to explore their potential in promoting health (Mazeas et al., 2022). Moreover, it is worth exploring whether remote ES can serve as an effective strategy to help vulnerable populations, who face barriers to physical activity, increase their exercise levels. Such interventions could potentially contribute to improving overall public health outcomes (Liang et al., 2023). This area warrants further investigation, particularly for populations with specific needs, such as individuals with disabilities or mental health challenges. However, unsupervised remote exercise should be implemented cautiously after appropriate medical assessment and may be most suitable for clinically stable older adults with lower levels of frailty.

Despite the promise of remotely delivered ES interventions, several key challenges in the current evidence base warrant attention. The lack of standardised ES protocols, particularly regarding programme design, duration, and progression, may contribute to inconsistent effects on balance. In addition, the optimal mode and intensity of digital guidance and supervision remain uncertain, given that remote ES typically relies on online platforms for instruction and monitoring. Integrating mobile applications and digital coaching may help strengthen delivery, improve adherence and acceptability, and support sustained health benefits. From a mechanistic perspective, although ES appears to improve muscle strength, its effects on balance—and the underlying neuromuscular pathways—are not yet well characterised. Future studies should incorporate biomechanical and neuromuscular assessments to better clarify these physiological processes.

## 5 Limitations

This study has several limitations. First, ES interventions and participant characteristics varied across trials, which may limit generalisability. Second, the number of eligible RCTs was small and sample sizes were modest; most trials also had short follow-up periods and limited blinding, which may increase the risk of bias. Third, adherence was largely self-reported, raising the possibility of reporting bias. Additionally, although pooled effects reached statistical significance, their practical relevance remains uncertain, and significant results came along with very limited confidence intervals. Future studies should incorporate clinically interpretable benchmarks (e.g., MCIDs) and functional endpoints to better contextualise the practical significance of ES. Finally, all included trials were assessed as having “some concerns” in risk of bias, indicating moderate methodological limitations; consequently, the overall certainty of the evidence is low, and the results should be interpreted with appropriate caution.

## 6 Conclusion

Remote exercise snacking appears effective in improving lower-limb muscle performance but shows limited impact on balance among healthy older adults. Intervention duration and the inclusion of progression are key determinants of efficacy. The delivery mode (e.g., written materials, video, or app-based platforms) and exercise type (e.g., bodyweight, Tai Chi, or combined formats) may influence the acceptability and feasibility of implementation.

## Data Availability

The original contributions presented in the study are included in the article/Supplementary Material, further inquiries can be directed to the corresponding authors.
