# Supplementary material for: Remote exercise snacking and fall-related functional outcomes in older adults: a systematic review including a meta-analysis
Source: Front Physiol. 2026 Feb 11;17:1709619. doi: 10.3389/fphys.2026.1709619 (PMC12932249; doi:10.3389/fphys.2026.1709619)
Supplement: Supplementary file 1 [file Table1.docx]

Supplementary Material

# Supplementary Figures and Tables

## Figure S1 Funnel plots.


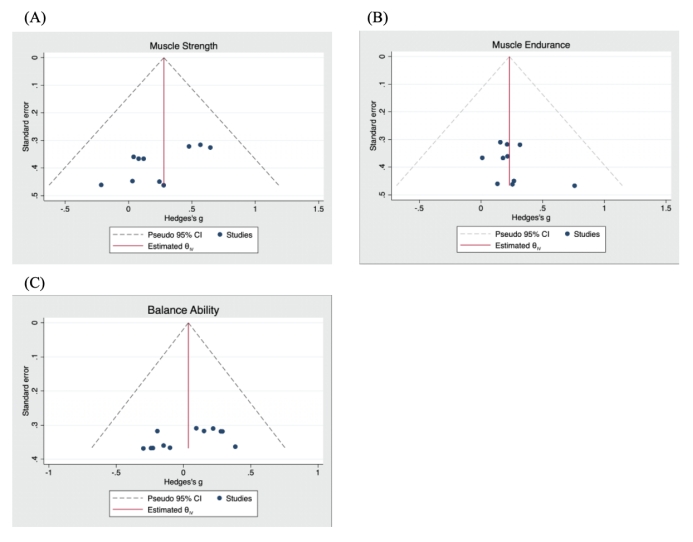
 Figure S1 Funnel plots.

## Table S1 Search Strategy

# Table S1 Search Strategy

| Database | Query | Results |
| --- | --- | --- |
| CINAHL | TX exercise snacks OR TX exercise snacking OR TX snacktivity OR TX movement breaks OR TX activity break OR TX physical activity breaks OR TX active break | 3820 |
| PubMed | ("Exercise snack*" OR "movement snack*" OR "snacktivity" OR "movement break" OR "physical activity break" OR "active break" OR "vigorous intermittent lifestyle physical activity" OR "VILPA")[All fields] | 1144 |
| Scopus | TITLE-ABS-KEY ( "Exercise snack*" OR "movement snack*" OR "snacktivity" OR "movement breaks" OR "physical activity breaks" OR "active breaks" OR "vigorous intermittent lifestyle physical activity" OR "VILPA" ) | 745 |
| Cochrane  Library | ("Exercise snack*" OR "movement snack*" OR "snacktivity" OR "movement breaks" OR "physical activity breaks" OR "active breaks" OR "vigorous intermittent lifestyle physical activity" OR "VILPA") in All Text (Word variations have been searched) | 366 |
| Web of  Science | ALL=("Exercise snack*" OR "movement snack*" OR snacktivity OR "movement break" OR "physical activity break" OR "active break" OR "vigorous intermittent lifestyle physical activity" OR "VILPA") | 339 |
| FMRS | ("Exercise snack"[ALL] OR "movement snack"[ALL] OR "snacktivity"[ALL] OR "movement break"[ALL] OR "physical activity break"[ALL] OR "active breaks"[ALL]) | 185 |

## Table S2 PRISMA Guideline

# Table S2 PRISMA Guideline

| **Section and Topic** | **Item #** | **Checklist item** | **Location where item is reported** |
| --- | --- | --- | --- |
| **TITLE** | | |  |
| Title | 1 | Identify the report as a systematic review. | Page1 title |
| **ABSTRACT** | | |  |
| Abstract | 2 | See the PRISMA 2020 for Abstracts checklist. | Page1 Abstract |
| **INTRODUCTION** | | |  |
| Rationale | 3 | Describe the rationale for the review in the context of existing knowledge. | Page2-3 Introduction |
| Objectives | 4 | Provide an explicit statement of the objective(s) or question(s) the review addresses. | Page2-3 Introduction |
| **METHODS** | | |  |
| Eligibility criteria | 5 | Specify the inclusion and exclusion criteria for the review and how studies were grouped for the syntheses. | Page3 Selection criteria |
| Information sources | 6 | Specify all databases, registers, websites, organisations, reference lists and other sources searched or consulted to identify studies. Specify the date when each source was last searched or consulted. | Page3 Data sources and search strategies |
| Search strategy | 7 | Present the full search strategies for all databases, registers and websites, including any filters and limits used. | Page3 Data sources and search strategies |
| Selection process | 8 | Specify the methods used to decide whether a study met the inclusion criteria of the review, including how many reviewers screened each record and each report retrieved, whether they worked independently, and if applicable, details of automation tools used in the process. | Page4 Data sources and search strategies |
| Data collection process | 9 | Specify the methods used to collect data from reports, including how many reviewers collected data from each report, whether they worked independently, any processes for obtaining or confirming data from study investigators, and if applicable, details of automation tools used in the process. | Page4 Data extraction and outcomes |
| Data items | 10a | List and define all outcomes for which data were sought. Specify whether all results that were compatible with each outcome domain in each study were sought (e.g. for all measures, time points, analyses), and if not, the methods used to decide which results to collect. | Page3-4 |
|  | 10b | List and define all other variables for which data were sought (e.g. participant and intervention characteristics, funding sources). Describe any assumptions made about any missing or unclear information. | Page3-4 |
| Study risk of bias assessment | 11 | Specify the methods used to assess risk of bias in the included studies, including details of the tool(s) used, how many reviewers assessed each study and whether they worked independently, and if applicable, details of automation tools used in the process. | Page5 Quality assessment |
| Effect measures | 12 | Specify for each outcome the effect measure(s) (e.g. risk ratio, mean difference) used in the synthesis or presentation of results. | Page4-5 Statistical analysis |
| Synthesis methods | 13a | Describe the processes used to decide which studies were eligible for each synthesis (e.g. tabulating the study intervention characteristics and comparing against the planned groups for each synthesis (item #5)). | Page4-5 Statistical analysis |
|  | 13b | Describe any methods required to prepare the data for presentation or synthesis, such as handling of missing summary statistics, or data conversions. | Page4-5 Statistical analysis |
|  | 13c | Describe any methods used to tabulate or visually display results of individual studies and syntheses. | Page4-5 Statistical analysis |
|  | 13d | Describe any methods used to synthesize results and provide a rationale for the choice(s). If meta-analysis was performed, describe the model(s), method(s) to identify the presence and extent of statistical heterogeneity, and software package(s) used. | Page4-5 Statistical analysis |
|  | 13e | Describe any methods used to explore possible causes of heterogeneity among study results (e.g. subgroup analysis, meta-regression). | Page4-5 Statistical analysis |
|  | 13f | Describe any sensitivity analyses conducted to assess robustness of the synthesized results. | Page4-5 Statistical analysis |
| Reporting bias assessment | 14 | Describe any methods used to assess risk of bias due to missing results in a synthesis (arising from reporting biases). | Page5 Quality assessment |
| Certainty assessment | 15 | Describe any methods used to assess certainty (or confidence) in the body of evidence for an outcome. | Page5 Quality assessment |
| **RESULTS** | | |  |
| Study selection | 16a | Describe the results of the search and selection process, from the number of records identified in the search to the number of studies included in the review, ideally using a flow diagram. | Fig. 1 and 3.1 Study selection |
|  | 16b | Cite studies that might appear to meet the inclusion criteria, but which were excluded, and explain why they were excluded. | Fig. 1 |
| Study characteristics | 17 | Cite each included study and present its characteristics. | Page5-6 and Table1. |
| Risk of bias in studies | 18 | Present assessments of risk of bias for each included study. | Page8 and Fig 2. |
| Results of individual studies | 19 | For all outcomes, present, for each study: (a) summary statistics for each group (where appropriate) and (b) an effect estimate and its precision (e.g. confidence/credible interval), ideally using structured tables or plots. | Page7-9 and Fig 3-5. |
| Results of syntheses | 20a | For each synthesis, briefly summarise the characteristics and risk of bias among contributing studies. | Page7-9 |
|  | 20b | Present results of all statistical syntheses conducted. If meta-analysis was done, present for each the summary estimate and its precision (e.g. confidence/credible interval) and measures of statistical heterogeneity. If comparing groups, describe the direction of the effect. | Page7-9 |
|  | 20c | Present results of all investigations of possible causes of heterogeneity among study results. | Page7-9 and Fig 3-5. |
|  | 20d | Present results of all sensitivity analyses conducted to assess the robustness of the synthesized results. | Page7-9 |
| Reporting biases | 21 | Present assessments of risk of bias due to missing results (arising from reporting biases) for each synthesis assessed. | Page7-9 |
| Certainty of evidence | 22 | Present assessments of certainty (or confidence) in the body of evidence for each outcome assessed. | Page7-9 |
| **DISCUSSION** | | |  |
| Discussion | 23a | Provide a general interpretation of the results in the context of other evidence. | Discussion |
|  | 23b | Discuss any limitations of the evidence included in the review. | Discussion |
|  | 23c | Discuss any limitations of the review processes used. | Discussion |
|  | 23d | Discuss implications of the results for practice, policy, and future research. | Discussion |
| **OTHER INFORMATION** | | |  |
| Registration and protocol | 24a | Provide registration information for the review, including register name and registration number, or state that the review was not registered. | Page3 Methods |
|  | 24b | Indicate where the review protocol can be accessed, or state that a protocol was not prepared. | Page3 Methods |
|  | 24c | Describe and explain any amendments to information provided at registration or in the protocol. | Page3 Methods |
| Support | 25 | Describe sources of financial or non-financial support for the review, and the role of the funders or sponsors in the review. | Page 11 |
| Competing interests | 26 | Declare any competing interests of review authors. | Page 11 |
| Availability of data, code and other materials | 27 | Report which of the following are publicly available and where they can be found: template data collection forms; data extracted from included studies; data used for all analyses; analytic code; any other materials used in the review. | Page 11 |

## Table S3 Subgroup analysis

Table S3. Subgroup analysis results regarding the effects of ES

| **Outcomes** | **Variables** | **No. of** | **SMD** | **P value** | **Test of heterogeneity** | |
| --- | --- | --- | --- | --- | --- | --- |
|  |  | **studies** | **(95%CI)** |  | **I2(%)** | **P value** |
| Strength | Duration (weeks) |  |  |  |  |  |
|  | ≤ 6weeks | 8 | 0.19 (-0.08~0.45) | 0.17 | 0 | 0.84 |
|  | > 6weeks | 2 | 0.58 (0.14~1.02) | 0.01 | 0 | 0.63 |
|  | Progression |  |  |  |  |  |
|  | Yes | 6 | 0.41 (0.12~0.7) | 0.01 | 0 | 0.52 |
|  | No | 4 | 0.11 (-0.26~0.47) | 0.57 | 0 | 0.98 |
| Endurance | Duration (weeks) |  |  |  |  |  |
|  | ≤ 6weeks | 8 | 0.22 (-0.04~0.48) | 0.1 | 0 | 0.98 |
|  | > 6weeks | 2 | 0.28 (-0.16~0.71) | 0.21 | 0 | 0.78 |
|  | Progression |  |  |  |  |  |
|  | Yes | 6 | 0.22 (-0.06~0.51) | 0.13 | 0 | 0.7 |
|  | No | 4 | 0.26 (-0.11~0.62) | 0.17 | 0 | 0.99 |
| Balance | Duration (weeks) |  |  |  |  |  |
|  | ≤ 6weeks | 4 | -0.02(-0.25~0.22) | 0.88 | 0 | 0.79 |
|  | > 6weeks | 2 | 0.15 (-0.16~0.45) | 0.35 | 0 | 0.56 |
|  | Progression |  |  |  |  |  |
|  | Yes | 3 | 0.16(-0.09~0.40) | 0.21 | 0 | 0.82 |
|  | No | 3 | -0.11(-0.39~0.18) | 0.46 | 0 | 0.76 |

# 2 Supplementary formula

# First, the difference in means was transformed via the following formula:


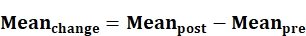


where Meanchange is the difference in means, Meanpre is the pre-intervention mean, and Meanpost is the post-intervention mean. Second, the standard deviation (SD) was calculated using the following formula based on the principles of the Cochrane Handbook for Systematic Reviews of Interventions:


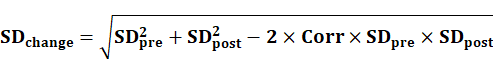


where SDchange is the SD of the difference in means, SDpre is the pre-intervention standard deviation, SDpost is the post-intervention standard deviation, Corr is the Pearson correlation coefficient, and following the recommendation of the Cochran handbook[20], we experiment with Corr = 0.80:


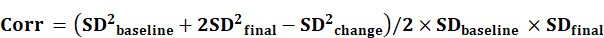


We referred to previous meta-analyses with similar results, with Corr values of 0.80[21], 0.85[22], and 0.89[23], respectively. After sensitivity analyses, we ultimately chose Corr = 0.85. Moreover, if the original study reported standard error (SE), the SD can be obtained from the SE of a mean by multiplying by the square root of the sample size:


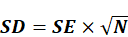


where N is the sample size.

**
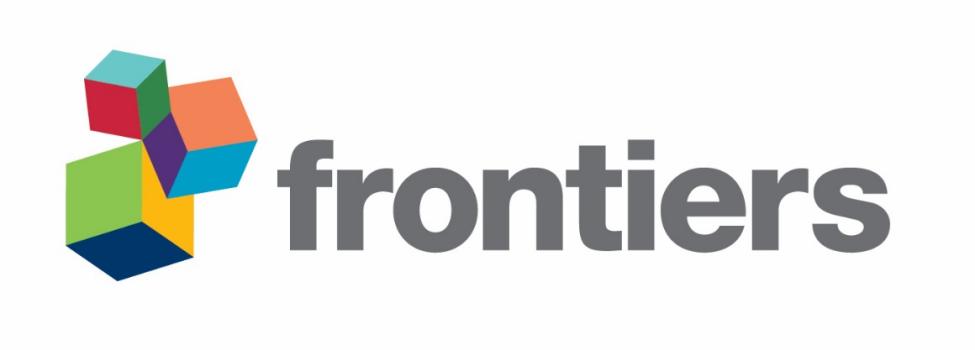
**
